# Effect of mechanical disruption on the effectiveness of three reactors used for dilute acid pretreatment of corn stover Part 1: chemical and physical substrate analysis

**DOI:** 10.1186/1754-6834-7-57

**Published:** 2014-04-09

**Authors:** Wei Wang, Xiaowen Chen, Bryon S Donohoe, Peter N Ciesielski, Rui Katahira, Erik M Kuhn, Kabindra Kafle, Christopher M Lee, Sunkyu Park, Seong H Kim, Melvin P Tucker, Michael E Himmel, David K Johnson

**Affiliations:** 1 National Renewable Energy Laboratory, 15013 Denver West Parkway, Golden, CO 80401, USA; 2 Department of Chemical Engineering and Materials Research Institute, Pennsylvania State University, University Park, PA 16802, USA; 3 Department of Forest Biomaterials, North Carolina State University, Raleigh NC 27695, USA

**Keywords:** Reactor, dilute acid pretreatment, biomass, cellulose properties, substrate accessibility, digestibility

## Abstract

**Background:**

There is considerable interest in the conversion of lignocellulosic biomass to liquid fuels to provide substitutes for fossil fuels. Pretreatments, conducted to reduce biomass recalcitrance, usually remove at least some of the hemicellulose and/or lignin in cell walls. The hypothesis that led to this research was that reactor type could have a profound effect on the properties of pretreated materials and impact subsequent cellulose hydrolysis.

**Results:**

Corn stover was dilute-acid pretreated using commercially relevant reactor types (ZipperClave^®^ (ZC), Steam Gun (SG) and Horizontal Screw (HS)) under the same nominal conditions. Samples produced in the SG and HS achieved much higher cellulose digestibilities (88% and 95%, respectively), compared to the ZC sample (68%). Characterization, by chemical, physical, spectroscopic and electron microscopy methods, was used to gain an understanding of the effects causing the digestibility differences. Chemical differences were small; however, particle size differences appeared significant. Sum-frequency generation vibrational spectra indicated larger inter-fibrillar spacing or randomization of cellulose microfibrils in the HS sample. Simons’ staining indicated increased cellulose accessibility for the SG and HS samples. Electron microscopy showed that the SG and HS samples were more porous and fibrillated because of mechanical grinding and explosive depressurization occurring with these two reactors. These structural changes most likely permitted increased cellulose accessibility to enzymes, enhancing saccharification.

**Conclusions:**

Dilute-acid pretreatment of corn stover using three different reactors under the same nominal conditions gave samples with very different digestibilities, although chemical differences in the pretreated substrates were small. The results of the physical and chemical analyses of the samples indicate that the explosive depressurization and mechanical grinding with these reactors increased enzyme accessibility. Pretreatment reactors using physical force to disrupt cell walls increase the effectiveness of the pretreatment process.

## Background

Currently, there is considerable interest in the conversion of lignocellulosic biomass to liquid fuels to provide substitutes for fossil fuels. Biomass is considered to be the only sustainable resource with the potential to deliver renewable fuels on a national scale [1,2]. However, the terrestrial plant cell wall has evolved into a complex structure, naturally recalcitrant to biological and chemical attack [3]. This high recalcitrance greatly impedes access of enzymes to biomass cellulose [4], thus increasing conversion costs. To date, no cost-effective process for converting biomass to liquid fuels has been adopted for commercial-scale biofuel production, as we still do not know how to efficiently overcome the barrier of biomass recalcitrance.

Pretreatments, conducted to reduce biomass recalcitrance, usually remove at least some of the hemicellulose and/or lignin in cell walls. Current pretreatment technologies operate with different chemistries at varying temperatures and for different reaction times [5]. Among the leading pretreatment technologies, dilute acid pretreatment has long been recognized as one of the more efficient pretreatments for producing materials that are more accessible to cellulase enzymes [6-8]. However, techno-economic analyses suggest that pretreatment accounts for a large part of the total capital investment of cellulosic biorefineries [9,10]. The choice of pretreatment technology is determined by the operational and capital equipment costs associated with that pretreatment technology balanced against the sugar yields that can be achieved. The hypothesis that led to this research was that reactor type could have a profound effect on the properties of pretreated materials and thus affect subsequent cellulose hydrolysis. Even at the same pretreatment severity, the choice of reactor type and its operational mode such as heating profile, solid mixing, pressure release, etc., could profoundly affect the physical structure of the pretreated substrates.

**Figure 1:**
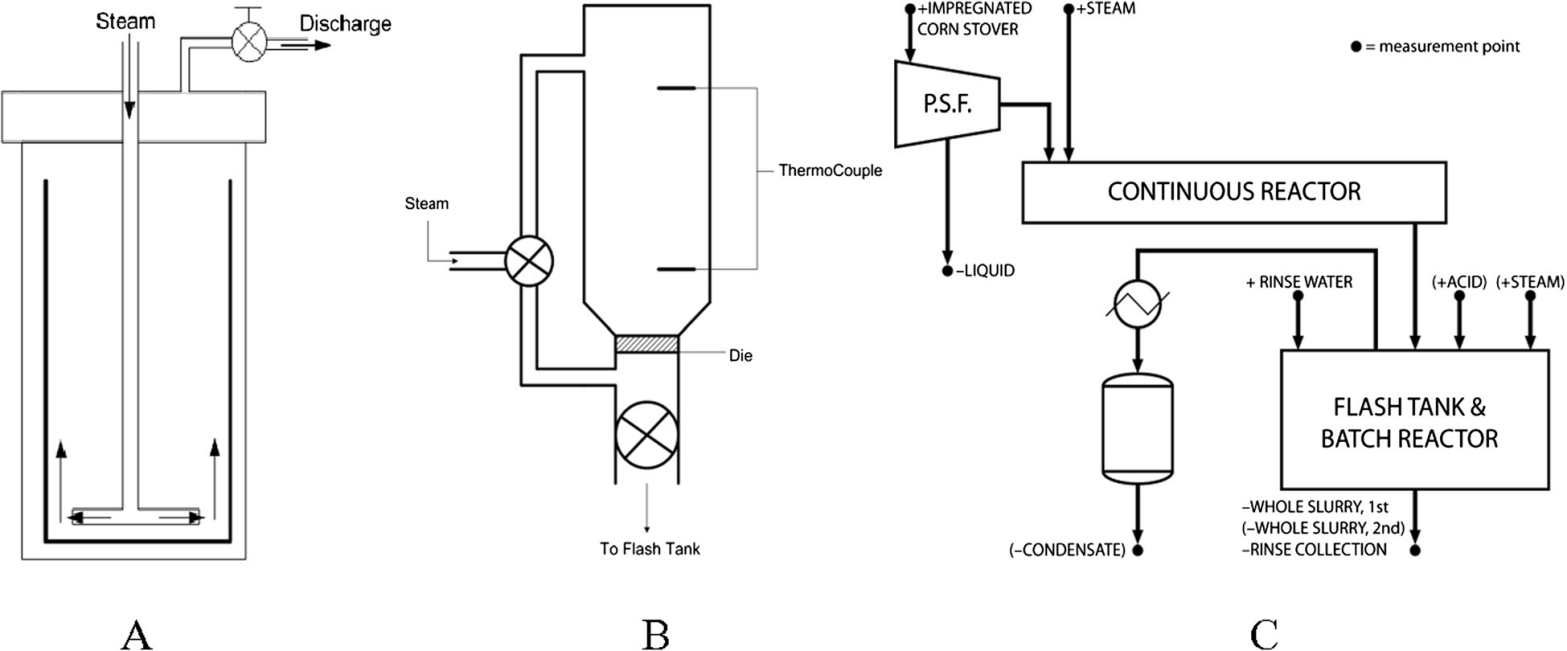
**Schematic diagrams of the three reactors. (A)** ZipperClave^®^ (ZC), **(B)** steam gun (SG) and **(C)** horizontal screw (HS) reactors.

In this study, pretreatment experiments have been conducted in three commercially relevant reactor types with widely different modes of operation. The diagrams for these three reactors are shown in Figure 1. The ZipperClave^®^ (ZC) and steam gun (SG) are batch reactors in which biomass is pretreated and discharged at the end of pretreatment, whereas the horizontal screw (HS) reactor is a screw-fed, plug flow reactor with continuous biomass feed. Not only is the mode of operation different for each reactor, but the pretreatment mechanism of each reactor is also different. For the SG reactor, pressure is explosively released at the end of pretreatment. With the ZC, steam pressure is released gradually through a globe valve into a heat exchanger. Decompression in the continuous HS reactor is rapid, with discharge of pretreated biomass to atmospheric pressure using two alternating ball valves. The differences in operation mode and biomass discharge have an impact on cellulose properties and accordingly on the enzymatic digestibility of the cellulose in the pretreated biomass.

In this study, corn stover feedstock was pretreated under the same temperature (160°C), residence time (5 minutes), and acid concentration (2.0 wt% sulfuric acid). Chemical and physical characterizations, as well as spectroscopic and imaging analyses were conducted on the pretreated corn stover to allow a side-by-side comparison of the effects of pretreatment in the three reactors. The mechanisms responsible for the differences in cellulose hydrolysis results are also discussed.

## Results

### Digestibility of pretreated corn stover samples

**Figure 2:**
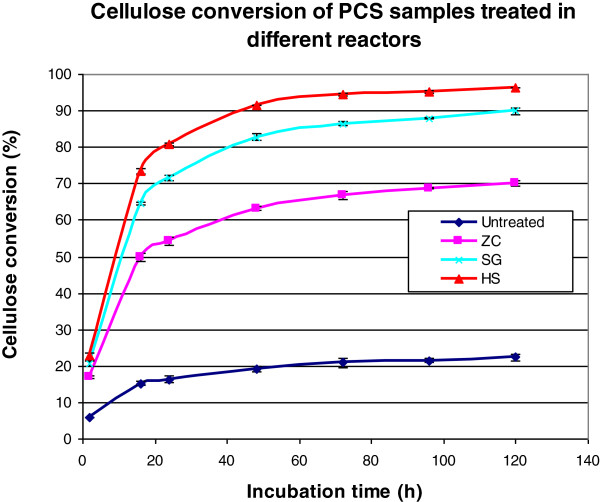
**Enzymatic digestion of acid pretreated corn stover with GC 220 (1% cellulose loading, enzyme: GC 220 20 mg/g cellulose, 50°C, pH 5.0, 130 rpm).** PCS, pretreated corn stover.

The pretreated corn stover samples from the three reactors were digested with a cellulase complex. Figure 2 shows the cellulose conversion for the three pretreated samples. All three samples exhibited classic digestion curves with a fast initial hydrolysis rate gradually leveling off before approaching maximum conversion. Compared to the ZC, the SG and HS reactor pretreated samples hydrolyzed much faster in the beginning and had higher final conversions at above 90% after 120 h, whereas the sample from the ZC only achieved 70% cellulose conversion. The properties of the pretreated samples were then examined to find explanations for the differences in digestibility of the samples.

### Composition of pretreated corn stover samples

Corn stover was dilute-acid pretreated in the ZC, SG and HS reactors as described above under the same nominal conditions: 160°C, 5 minutes, and 2.0 wt% sulfuric acid. The compositions of the pretreated samples from each reactor are listed in Table 1. Generally, the compositions of the corn stover samples pretreated in the three reactors were similar. Among the three reactors, the HS and SG reactors had more xylan removal than the ZC reactor, as shown in Table 2.

**Table 1:** Composition of pretreated corn stover

	**Total ash, %**	**Extractives, %**	**Lignin, %**	**Glucan, %**	**Xylan, %**	**Galactan, %**	**Arabinan, %**	**Acetyl, %**	**Mass closure, %**
Untreated	5.8	12.3	12.3	34.0	22.0	1.6	3.1	2.9	94.0
ZC	6.2	0.0	24.1	53.3	9.0	0.7	2.2	1.5	97.0
SG	6.5	0.0	24.0	60.2	4.8	0.7	2.1	0.6	98.9
HS	9.2	0.0	25.9	57.4	3.2	0.1	0.4	0.6	96.8

*ZC*, ZipperClave^®^; *SG*, steam gun; *HS*, horizontal screw.

**Table 2:** Xylan removal from corn stover by acid pretreatment in the three reactors

**Reactor**	**Xylan removal, %**
ZC	79
SG	88
HS	93

The potential error in determining xylan removal was estimated at < ±1%. *ZC*, ZipperClave^®^; *SG*, steam gun; *HS*, horizontal screw.

### Changes in biomass components as measured by ^13^C cross polarization/magic-angle spinning (CP/MAS) solid-state nuclear magnetic resonance (NMR) spectroscopy

**Figure 3:**
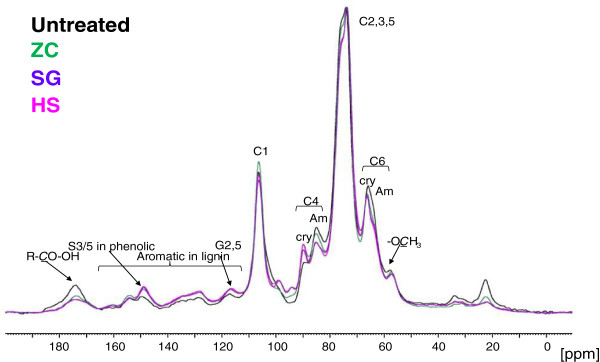
**13C cross polarization/magic-angle spinning (CP/MAS) solid-state nuclear magnetic resonance (NMR) spectra of untreated and pretreated corn stover from three reactors.** Spectra were normalized to the cellulose peak at 72 ppm. ZipperClave^®^; SG, steam gun; HS, horizontal screw.

The ^13^C CP/MAS solid-state NMR spectra of untreated and pretreated corn stover samples are shown in Figure 3. All peaks were assigned from references in the literature [11-13].

The cellulose region (60 to 110 ppm) is typical of pretreated corn stover samples. The crystallinity of the cellulose cannot be assessed as with pure celluloses because of overlap from peaks in this region due to the xylan and lignin that remain in the samples. Overall, the NMR spectra of these samples closely resembled that of the unpretreated whole corn stover starting material. There were a few relatively small differences in the spectra, mostly indicating changes in lignin structure and hemicellulose content.

Peaks in the 165 to 185 ppm region were assigned to carbonyl groups. These peaks are mainly derived from the acetyl groups in hemicelluloses. As the hemicellulose was rapidly solubilized during dilute acid pretreatment, acetyl groups in the hemicellulose were removed. Compared to corn stover pretreated in the SG and HS reactors, slightly more carbonyls remained in the ZC pretreated sample. This corresponds to the higher level of residual xylan remaining in pretreated solid from the ZC and agrees with the compositional analyses.

In the aromatic peak region corresponding to lignin (110 to 165 ppm), phenolic S3/5 peaks at 149 ppm increased for all three samples, indicating that β-*O*-4 ether linkages in lignin were cleaved during pretreatment and free phenolic hydroxyl groups were produced. The three pretreated samples had almost the same lignin spectrum except that the ZC pretreated sample had slightly more non-phenolic (154 ppm) and fewer phenolic (149 ppm) groups in the lignin structure, suggesting that pretreatment in the ZC was milder and less destructive to lignin than pretreatment in the other two reactors.

### Particle size of pretreated corn stover samples

**Figure 4:**
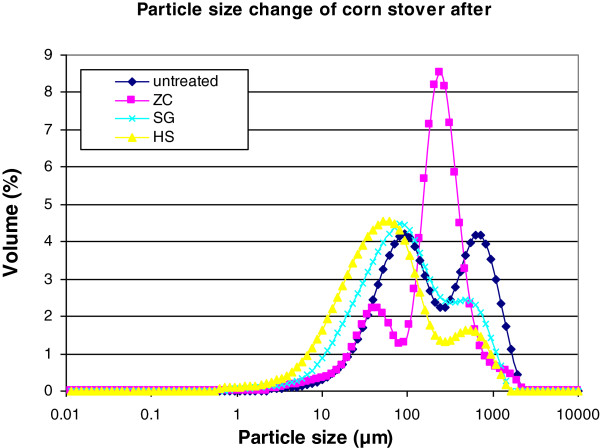
**Particle-size distribution of pretreated corn stover samples.** Each data point is the average of three replicates. ZipperClave^®^; SG, steam gun; HS, horizontal screw.

The particle sizes of the pretreated corn stover samples were measured by laser diffraction. These analyses showed a bimodal distribution of particles with some particles in the 10 to 100 μm range and others in the 100 to 2000 μm range (Figure 4). After pretreatment, the particle size distribution of all the samples shifted to smaller particles. For the SG and HS reactor samples, not only did the fraction of large particles decrease, but also the distribution of the small particles shifted to a smaller size. The corn stover pretreated in the ZC had a much larger fraction of large particles than the other two reactors. Overall, there was a significant decrease in the mean particle sizes of the samples due to pretreatment, from the mean particle size of the untreated corn stover (343 μm) to the ZC (245 μm), SG (187 μm) and the HS reactor (134 μm) pretreated materials. Compared to acid pretreatment without explosive decompression, pretreatment in the SG and HS reactors appeared to be more destructive to the integrity of the biomass, resulting in smaller particle sizes. A further decrease in particle size was obtained with the HS reactor possibly due to the grinding effect of the screw inside the reactor.

Particle size is considered one of the structural features that can have the greatest affect on biomass enzymatic digestibility. Our results showed both acid pretreatment with and without explosive decompression decreased biomass particle size. Increasing cellulase adsorption and cellulose digestibility by decreasing particle size has previously been reported [14-16]. The decrease in particle size is indicative of a change in the physical structure of the biomass and could result in an increase in digestibility by decreasing mass transfer resistance or by increasing the exposed external surface area of the biomass particles.

### Cellulose degree of polymerization (DP)

Cellulose DP is thought to be one of the many factors that influence cellulose hydrolysis [17,18]. In this study, pretreatment in the different reactors produced little difference in cellulose DP. The DP of all three samples dropped from 7,200 to about the same level 1,600 to approximately 1,800. It is well-recognized that the molecular weight of cellulose decreases when treated with acid [19-22]. Our results show that the decrease in cellulose DP was not affected by the different operational modes of the reactors, and it appears unlikely that changes in cellulose DP can explain the differences in digestibility of these samples.

### Cellulose crystal structure as measured by sum-frequency generation (SFG) vibration spectroscopy

SFG is a non-linear optical process that takes place only when an optical medium without inversion symmetry is irradiated with high-intensity laser pulses [23]. The crystalline cellulose in microfibrils has certain vibration modes that meet this noncentrosymmetry requirement; thus, cellulose in biomass is SFG-active. Hemicellulose and lignin are amorphous and therefore not SFG-active. Using this principle, crystalline cellulose in lignocellulosic biomass can be selectively detected by SFG vibration spectroscopy without spectral interference from non-cellulosic components [24]. The SFG signal intensity is also sensitive to spatial arrangement or distribution of crystallites over the optical coherence length of the SFG process in the sample [25]. Thus, SFG can provide information about the cellulose crystal structure and crystalline cellulose arrangement in biomass without the requirement for isolation or purification of the cellulose.

**Figure 5:**
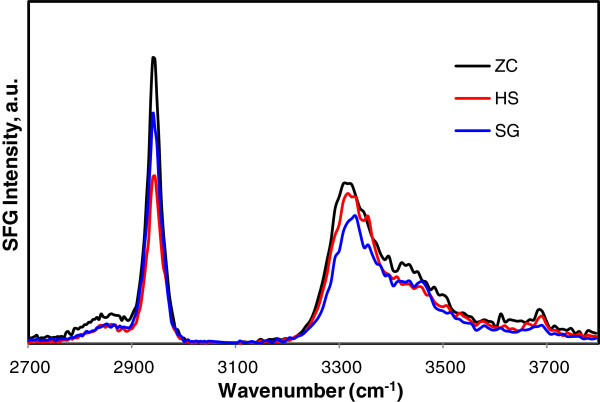
**Sum-frequency generation (SFG) spectra of pretreated corn stover samples.** ZipperClave^®^; SG, steam gun; HS, horizontal screw.

Figure 5 compares the SFG vibrational spectra of the pretreated corn stover samples from the three different reactors. All samples show the same peaks: a weak peak at 2,850 cm^-1^ and a strong peak at 2,944 cm^-1^ in the C-H stretch region, and a peak at 3,320 cm^-1^ with a shoulder at 3,450 cm^-1^ in the O-H stretch region. The 2,850 cm^-1^ and 2,944 cm^-1^ peaks are assigned to symmetric and asymmetric CH_2_ stretch vibrations, respectively, of the cellulose Iβ crystal [24]. The 3,320 cm^-1^ peak is mainly due to the O-H groups involved in intra-chain hydrogen-bonding interactions in the cellulose Iβ crystal. The main difference among the samples is the CH_2_/OH intensity ratio. It is especially noted that the CH_2_/OH ratio is small for the HS reactor sample (1.1 ± 0.1) compared to the ZC (1.7 ± 0.4) and SG reactor (1.7 ± 0.5) samples. This could be related to variations in the ordering of cellulose microfibrils in the pretreated cell wall. The net dipole of the 3,320 cm^-1^ OH stretch mode directs along the cellulose chain, whereas the dipole of the 2944 cm^-1^ CH_2_ stretch mode is assumed to be perpendicular to the cellulose chain direction [24]. The coherence of the polar ordering of vibration modes perpendicular to the fibril direction would be more sensitive to the separation distance or relative orientation between fibrils than that of vibration modes parallel to the fibril direction. Thus, the decrease in CH_2_ intensity for the HS reactor sample without a significant change in OH intensity, compared to the other samples, would suggest more separation or randomization of cellulose microfibrils in this sample compared to those produced in the other reactors.

### Substrate accessibility as measured by the Simons’ staining technique

**Figure 6:**
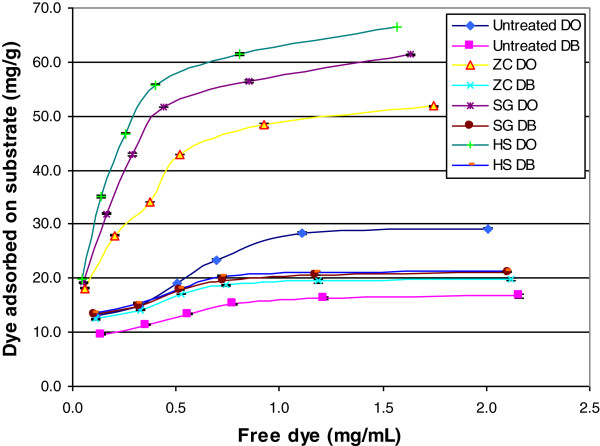
**Adsorption isotherms of dyes on pretreated samples.** ZipperClave^®^; SG, steam gun; HS, horizontal screw; DB, direct blue; DO, direct orange.

**Figure 7:**
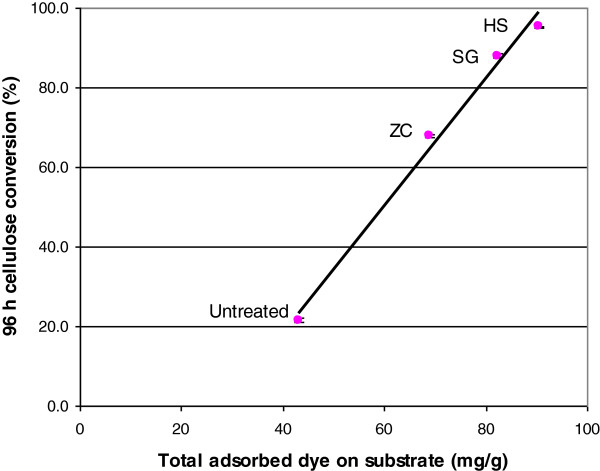
**Correlation of the orange/blue (O/B ratio) with cellulose conversion (after 96 h) for the untreated and pretreated samples.** ZipperClave^®^; SG, steam gun; HS, horizontal screw.

The Simons’ staining technique has been used for measuring biomass accessibility/ reactive surface area and has shown success in estimating a given pretreated substrate susceptibility to hydrolysis by cellulases [26,27]. The adsorption isotherm of each dye was obtained by plotting the free-dye concentration in the solution versus bound dye on the fiber (Figure 6). Analysis of the adsorption isotherm of dyes was performed using a Langmuir isotherm model, which has previously been used to describe absorption of cellulase to cellulose [28,29]. *B*_max_, the maximum amount of direct blue (DB) or direct orange (DO) dye adsorbed to a cellulosic substrate, calculated using Equation 3, as described in the Methods section, was used to calculate the orange/blue (O/B) ratio, which is used to estimate the substrate accessibility of lignocellulosic samples [30,31]. Compared to untreated corn stover, all three pretreated samples showed a higher O/B value (Table 3) than the untreated sample, reflecting increased accessibility of the cellulose. In addition, the SG and HS reactor pretreated samples exhibited higher O/B ratios than the sample pretreated in the ZC. A good correlation was found between the O/B ratio and cellulose conversion (after 96 h) for each sample (Figure 7). These results indicate that reactor operation can produce substantially different levels of cellulose accessibility leading to significant differences in cellulose digestibility.

**Table 3:** Adsorption of dyes and cellulose conversion of substrates

**Sample**	**Maximum adsorbed orange dye, mg/g substrate**	**Maximum adsorbed blue dye, mg/g substrate**	**Orange/blue ratio**	**Cellulose conversion at 96 h, %**
Untreated	26.4	16.8	1.57	21.5
ZipperClave^®^	49.3	19.6	2.51	67.8
Steam gun	61.7	20.5	3.01	88.0
Horizontal screw	69.4	20.9	3.32	95.2

### Cell wall ultrastructure as measured by transmission electron microscopy (TEM)

**Figure 8:**
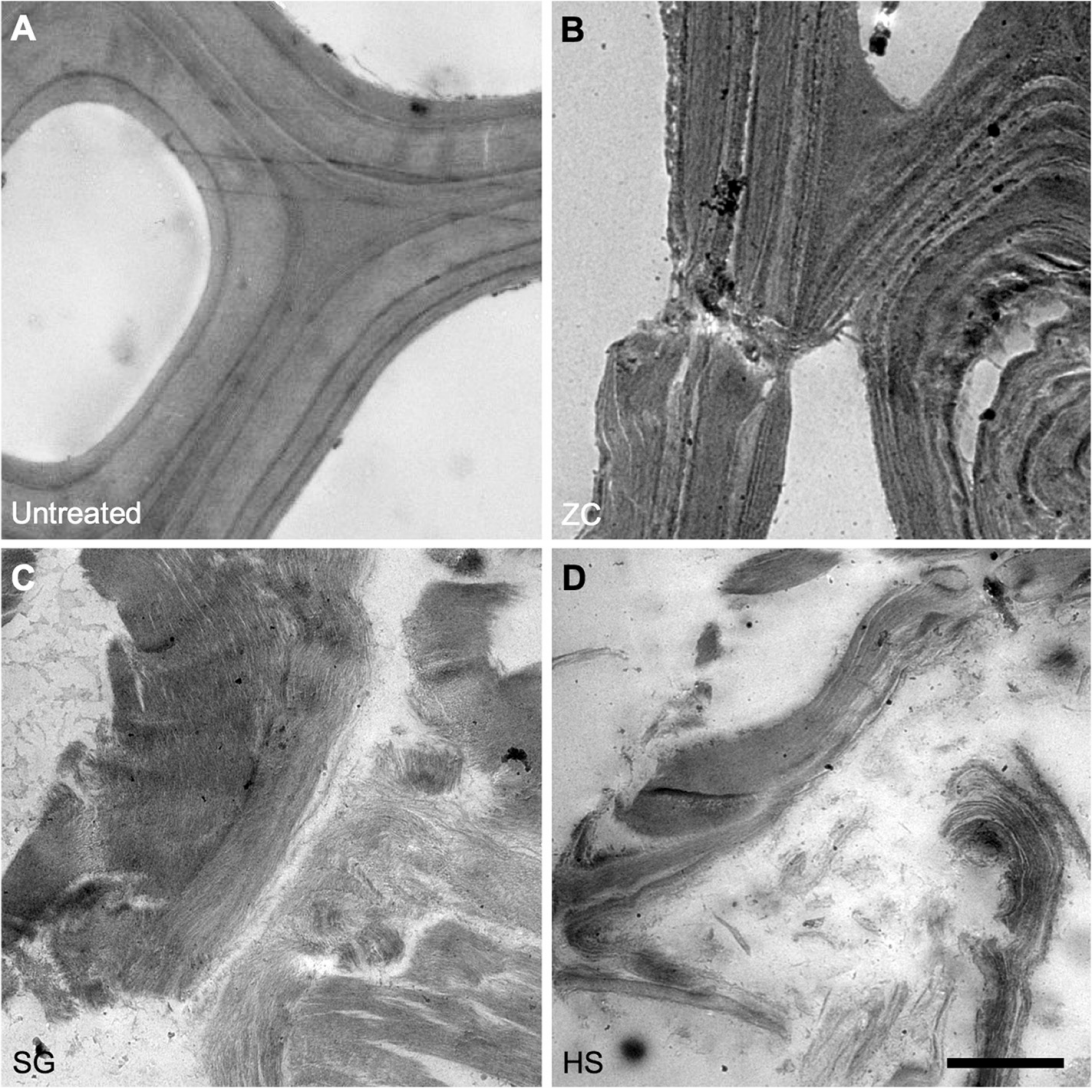
**TEM micrographs display varying degrees of delamination within cell walls. (A)** Untreated control **(B)** ZipperClave^®^**(C)** steam gun **(D)** horizontal screw reactor. The steam gun and horizontal screw samples display extensive fragmentation and delamination of the cell walls. (scale bar = 5 μm).

The differences in cell wall ultrastructure revealed by TEM were quite striking. The ZC samples displayed the characteristic evidence of delamination and relocalization of lignin evidenced by a cell-wall banding pattern with increasing contrast compared to the control (Figure 8A and B). We have become accustomed to seeing delamination within the S2 layer and between the S2 and S3 layers, but the extensively and finely delaminated cell walls seen in the SG and HS reactor samples are impressive (arrows, Figure 8C and D). In addition to dislocation between adjacent cells and delamination between the major cell wall layers, the SG and HS reactors generate a micro-fibrillation of the cell wall. Across large areas of the wall it appears that every lamella has been separated and individual or small bundles of microfibrils can be seen.

## Discussion

Among the three reactors, the HS reactor produced a solid substrate that exhibited the highest cellulose digestibility. Results from analysis of the chemical and physical structural features of the pretreated samples indicate the reasons for the higher digestibility observed for samples from the horizontal reactor. The results also help us gain a better understanding of the general factors governing biomass recalcitrance to enzymatic hydrolysis.

The limiting factors that affect enzymatic hydrolysis of biomass are usually divided into two groups: one group is related to the chemistry and structure of the substrate and the other is related to the mechanisms and interactions of the cellulase enzymes with the substrate. There are several structural features considered to be involved in biomass recalcitrance including cellulose crystallinity, cellulose DP, substrate porosity, particle size, and surface area. From the results of this study, it appears that dilute-acid pretreatment with and without explosive decompression made little difference to cellulose DP. In this study, there were slightly higher levels of xylan removal in the corn stover pretreated in the SG and HS reactor, although all pretreated materials had high levels of xylan removal (that is, close to or above 80%). Jeoh and coworkers [4] previously showed that xylan removal above 80% has little influence on cellulose digestibility in pretreated biomass.

The physical property that was most affected by using different reactors was particle size. The decreased biomass particle size resulted in increased external surface area that could increase accessibility of the enzymes to the substrate; however, this is just one indicator that the operation of the reactors produced greater enzyme accessibility. The SG and HS reactors add a mechanical component to pretreatment severity that affects particle size; however, this is not the only effect of the mechanical action of these reactors. TEM imaging shows that there is substantial delamination and fibrillation in the SG and HS reactor pretreated samples that are likely caused by the explosive decompression at the exits to these reactors. Mechanical shearing of the samples is also caused by the grinding action of the mixing screws inside the HS reactor. SFG vibration spectra indicate increased inter-fibrillar spacing or randomization of the cellulose microfibrils, which would increase the accessibility of enzymes to cellulose microfibril surfaces. The results from the Simons’ stain technique confirm that pretreatment with the SG and HS reactor significantly increased the accessibility of the cellulose in the substrates obtained with these reactors. Overall these techniques indicate that the operation of the reactors resulted in significant differences in the internal and external surface areas of the pretreated biomass particles, leading to differences in accessibility.

Substrate accessibility or available reactive area is a collective reflection of biomass structural features, which include but are not limited to cellulose crystallinity, biomass particle size, and pore volume. This parameter is intrinsically difficult to define and quantify. It is well-recognized that cellulose, hemicellulose and lignin are closely cross-linked in natural plant cell-walls, forming a very tightly packed structure. This dense macromolecular matrix coupled to highly ordered crystalline cellulose limit enzyme accessibility. Indeed, some researchers have indicated that substrate accessibility is a key factor affecting substrate-enzyme interactions, limiting the efficiency of enzymatic hydrolysis [4,17,32-35].

The differences in cellulose digestibilities of the samples pretreated in the three reactors studied here demonstrate the importance of the mechanical aspect to the operation of these three reactors. Differences in cellulose digestibility may be attributed to enzyme accessibility, which is substantially increased by the rapid decompression in the SG and HS reactor and by the grinding action of the mixing screws in the HS reactor. The comparatively slow decompression of the ZC does not produce the extensive changes in cell wall structure observed in samples produced in the other two reactors. Therefore, when designing pretreatment reactors, consideration should be given to utilizing mechanical force that could beneficially disrupt biomass cell walls during pretreatment.

## Conclusions

Dilute-acid pretreatment of corn stover using three different reactors under the same nominal conditions gave samples with very different digestibility, although chemical differences were small. Particle size was the one physical property that appeared significantly different in the pretreated samples. SFG vibrational spectra and Simons’ staining indicated increased cellulose accessibility in the SG and HS samples. TEM images showed these samples to be more fibrillated. It is likely that the explosive depressurization and mechanical grinding with these reactors caused increased enzyme accessibility. Pretreatment reactors using physical force to disrupt cell walls increase the effectiveness of the pretreatment process.

## Methods

### Biomass feed stocks

The corn stover used in this study was harvested from the Kramer farm in Wray, CO, USA. The feedstock was milled to ¼ inch prior to dilute acid pretreatment.

### Dilute acid pretreatment of biomass in different reactors

Seven 15-kg batches of corn stover feedstock were impregnated with dilute sulfuric acid (2.0 wt%) for 3 h using a recirculating bath, and then combined and mixed by a modified method of coning and quartering [36,37]. The mixed impregnated feedstock was dewatered using a horizontal tapered screw-press to 60% total solids prior to pretreatment, and then mixed again by coning and quartering. The pretreatment conditions for all three reactors (Figure 1) were the same: 160°C, 5 minutes and 2.0 wt% sulfuric acid. The pretreated slurry was washed with de-ionized (DI) water six times before enzymatic hydrolysis.

### ZC reactor

Pressed dilute-acid impregnated feedstock (160 g) was inserted into the 4-L ZC^®^ vertically stirred reactor (Autoclave Engineers, Erie, PA, USA). Steam was directly injected into the bottom of the reactor through ports in a *rotary*-*plow* type of agitator and constant temperature was achieved by controlling the steam pressure in the reactor. The ZC reactor is also equipped with an electrical heating blanket set at reaction temperature to lessen steam condensation due to heat losses through the reactor wall. The contents within the ZC reactor typically reached reaction temperature within 5 to 10 s of starting the steam flow as measured by two thermocouples, one inserted into the bottom and one near the middle of the reactor. At the end of pretreatment, the steam pressure was slowly released through a condenser over a period of 15 to 30 s to lessen boil-over, then the pretreated solids were sealed in a plastic freezer tub and stored at 4°C for later analysis.

### SG reactor

The 4-L National Renewable Energy Laboratory (NREL) SG reactor is constructed of Hastelloy C-22 for corrosion resistance. A steam jacket, thick insulation, and temperature-controlled electrical heating bands limit heat loss to the environment, thereby reducing steam condensation inside the reactor during pretreatment. The NREL SG was loaded with 500 g of sulfuric acid-impregnated and pressed corn stover that was quickly heated (approximately 5 to 10 s) via direct steam injection to the desired reaction temperature as measured by two thermocouples. At the end of the residence time, the pretreated solids were explosively discharged into a nylon HotFill^®^ bag inside a 200-L flash tank. The bags were removed from the flash tank and stored at 4°C until ready for analysis.

### HS reactor

The single-screw continuous HS pretreatment reactor fabricated by Metso Paper Inc. (Norcross, GA, USA; formerly Sunds Defibrator), was constructed of Hastelloy C2000. The reactor was heated via direct steam injection. The reactor is also equipped with steam jackets that reduce heat loss from the barrel of the reactor to the environment. A nominal throughput rate of 200 kg dry feedstock/day was used to perform continuous steady-state pretreatment. Acid-impregnated biomass and steam were continuously fed to the barrel of the reactor by a plug screw feeder and discharged at the other end to atmospheric pressure through two alternating ball valves. Pretreated slurry was collected from the flash tank and stored at 4°C for later analysis.

### Composition analysis of biomass sample

The compositions of raw and pretreated corn stover samples were measured in duplicate using the standard NREL methods for determining biomass carbohydrates, acid insoluble lignin, ash and acetate content [38]. The standard errors for these analyses were below the 1.5 wt%.

### Enzyme digestions

The commercial enzyme formulation GC 220 (Genencor/Danisco) was used to digest the corn stover samples. Digestions were performed in 125-mL Erlenmeyer flasks containing 50 mM citrate buffer, pH 4.8, at a biomass loading of 1.0% glucan (w/v). Digestion conditions were 130 rpm at 50°C. The cellulase enzyme was loaded at 20 mg protein per g of cellulose. Samples were taken periodically and analyzed by high performance liquid chromatography (HPLC) for cellobiose and glucose. Cellulose conversion was defined as the percentage of glucose and cellobiose released compared to the theoretical maximum.

### Determination of cellulose degree of polymerization (DP)

The molecular weight distribution of cellulose in pretreated corn stover was determined by size-exclusion chromatography (SEC). The procedure was modified from prior published methods [39,40]. Samples were first carbanilated so that cellulose would dissolve in the SEC eluant tetrahydrofuran (THF), and 10 mg of a vacuum dried sample was placed into a 5-mL reaction vial and 2 mL of dry pyridine and 1.2 mL of phenyl-isocyanate were added. The reaction vial was kept at 70°C for 24 h to complete the reaction. Methanol (1.2 mL) was added to the reaction mixture to react with the excess phenyl-isocyanate at the end of the reaction. The carbanilated cellulose was then precipitated in 26 mL of methanol/water (7:3, v/v) and the precipitate was washed twice with methanol/water (26 mL) before being dissolved in THF (20 mL).

SEC was performed using five columns (PLgel 10^3^, 10^4^, 10^5^, 10^6^ and 10^7^ Ǻ) to cover the broad range in molecular weight of the cellulose samples. A calibration curve was obtained using narrow polystyrene standards of known molecular weight to convert retention time into molecular weight. Consequently, all molecular weights determined in this work are not absolute, but are only relative to the calibration curve. The conditions for the chromatography were as follows: flow rate 1.0 mL/minute, UV detector wavelength 235 nm, injection volume 50 μL, and column temperature 25°C. Cellulose DP was calculated by dividing the apparent molecular weights by 519 (molecular weight of a repeating unit of a carbanilated cellulose with the degree of substitution of 3.0).

### Determination of particle size

The particle size of biomass samples was measured using laser diffraction on a Mastersizer 2000 with the Hydro 2000G module (Malvern Instruments, Worcestershire, UK). The instrument measures particle sizes over the range from 0.02 to 2,000 μm in a recirculating liquid suspension. For the analysis, 0.05 to 0.2 g of each cellulose sample was dispersed in water in a 15-mL centrifuge tube. Thereafter, individual dispersed samples were vortex mixed and transferred to the Hydro 2000G module that contained 0.8 to 1.0 L of deionized water, with a stirrer setting of 600 rpm and a pump setting of 1,250 rpm. After a 30-s delay, three 15-s readings (30 s apart) of the circulating samples were acquired and averaged. The volume-weighted mean value was used to represent the mean particle diameter (MPD). Each sample was run in triplicate and MPD is shown as the average of the triplicates.

### ^13^C CP/MAS solid-state NMR analysis

The high-resolution ^13^C CP/MAS solid-state NMR spectra were recorded on a Bruker Avance 200 MHz spectrometer at 4.7 T with a 7 mm BL probe (HP WB 73A MAS 7 BL CP VTN), operating at 50.13 MHz for ^13^C at room temperature [41]. The spectral acquisition parameters were: spinning speed 7,000 Hz, contact pulse 2 ms, acquisition time 32.8 ms, delay between pulses 1 s, and 50,000 scans. The adamantane peak was used as an external reference (δ_C_ 38.3 ppm). Chemical shifts (δ) were given in δ values (ppm).

### SFG vibration spectroscopy

Pretreated corn stover samples were analyzed with SFG vibration spectroscopy [24]. The 1,064 nm 27 ps laser pulse with a repetition rate of 10 Hz from an Nd:YAG laser (EKSPLA) was transformed to a 532 nm laser pulse through a frequency-doubling crystal. A tunable 2.3 to 10 μm infrared laser pulse was generated through optical parameter generation/amplification processes of the Nd:YAG laser outputs. The p-polarized infrared beam and s-polarized visible beams were temporally and spatially overlapped on the pressed pellet sample. The incidence angles of the infrared and visible beams were 56° and 60° with respect to surface normal, respectively. The SFG signal was detected in the reflection geometry with a beam collimator to increase the signal collection efficiency. A monochromator was used for filtering the SFG signal (s-polarized) which was recorded with a photomultiplier. SFG spectra were taken at 4 cm^-1^ intervals in the C-H stretch vibration region (2,700 to 3,050 cm^-1^) and 8 cm^-1^ intervals in the O-H stretch vibration region (3,100 to 3,800 cm^-1^). Each data point was an average of 100 laser shots, with SFG intensity normalized over IR and visible input laser intensities at each shot. Each spectrum shown in this paper represents the average of spectra from 10 different locations on the sample pellet.

### Simons’ staining for measurement of pore volume

The Simons’ staining technique was employed to quantify the total surface area/pore volume of corn stover samples following the method of Chandra [26]. Two direct dyes, Pontamine Fast Sky Blue 6BX and Pontamine Fast Orange 6RN (Pylam Products, Garden City, NY, USA) were used for this measurement. Biomass samples (100 mg) were weighed into six 15-mL centrifuge tubes, and 1.0 mL of PBS (pH 6.8, 0.1 M) was added to each tube. A 1:1 mixture of DB (10 mg/mL) and DO (10 mg/mL) solution was added to each tube in a series of increasing volumes (0.25, 0.50, 0.75, 1.0, 1.5, and 2.0 mL for each dye solution). Distilled water was added to each tube to make a final volume of 10.0 mL. The tubes were kept at 70°C for 6 h with shaking at 200 rpm. After incubation, the tubes were centrifuged at 10,000 rpm for 5 minutes and the supernatant was measured for absorbance at 455 nm and 624 nm on a UV-visible spectrophotometer (Beckman Coulter DU 800, Brea, CA, USA). The concentration of free DB or DO dye in the supernatant, *C*, was determined using the following two equations (Lambert-Beer law) [30], where *A* is the absorbance of the mixture at 455 or 624 nm, *ϵ* is the extinction coefficient of DB or DO at the respective wavelength, and *L* is the path length (1 cm in this study):

(1) $$A_{455}\;=\;\epsilon_{B455}\;LC_{B}\;+\;\epsilon_{O455}\;LC_{O}$$

(2) $$A_{624}=\epsilon_{B624}\;LC_{B}+\epsilon_{O624}\;LC_{O}$$

A Langmuir isotherm was used to describe adsorption of the dyes to cellulose. The maximum amount of DB or DO dye adsorbed to cellulosic substrate can be calculated using the following equation:

(3) $$B=\frac{B_{\text{max}}\left[C\right]}{\mathrm{Kd}+\left[C\right]}$$

where B (mg/g substrate) is the amount of dye bound to the substrate, [*C*] (mg/mL) is the free dye concentration in the solution, *B*_max_ is the maximum amount of dye bound to the cellulosic substrate, and *Kd* is the dissociation constant for the dye-substrate complex at adsorption equilibrium.

### TEM

Thin (60 nm) resin-embedded sections were positioned on 0.5% Formvar-coated copper slot grids (SPI Supplies, West Chester, PA, USA). Grids were post-stained for 6 minutes with 2% aqueous uranyl acetate and for 6 minutes with 1% aqueous KMnO_4_ to selectively stain for lignins. Images were taken with a 4 mega-pixel Gatan UltraScan 1000 camera (Gatan, Pleasanton, CA, USA) on a FEI Tecnai G2 20 Twin 200 kV LaB6 TEM (FEI, Hilsboro, OR, USA).

## Abbreviations

CP/MAS: cross polarization/magic-angle spinning; DB: direct blue; DO: direct orange DP, degree of polymerization; HS: horizontal screw; MPD: mean particle diameter; NMR: nuclear magnetic resonance; NREL: National Renewable Energy Laboratory; PBS: phosphate-buffered saline; SEC: size exclusion chromatography; SFG: sum-frequency generation; SG: steam gun; TEM: transmission electron microscopy; THF: tetrahydrofuran; ZC: ZipperClave^®^.

## Competing interests

The authors declare no competing interests.

## Authors’ contributions

XC, EK and MPT performed the biomass pretreatments. RK performed the NMR analysis. KK, CML, SP and SHK were involved in the performance of the SFG analysis. WW performed the enzyme digestions, dye adsorption analysis and with assistance from DKJ the cellulose DP measurements. PNC and BSD performed the microscopy analysis. MEH helped conceive the study and revise the manuscript. WW conceived the study and drafted the original manuscript. DKJ advised on the design and progress of the experimentation and helped draft and revise the manuscript. All authors read, edited, and approved the final manuscript.

## References

[B1] Farrell AE Plevin RJ Turner BT Jones AD O’hare M Kammen DM Ethanol can contribute to energy and environmental goals Science 2006 311 506 10.1126/science.1121416 16439656

[B2] Lynd LR Larson E Greene N Laser M Sheehan J Dale BE McLaughlin S Wang M The role of biomass in America’s energy future: framing the analysis Biofuels Bioprod Biorefin 2009 3 113 123 10.1002/bbb.134

[B3] Himmel ME Ding SY Johnson DK Adney WS Nimlos MR Brady JW Foust TD Biomass recalcitrance: engineering plants and enzymes for biofuels production Science 2007 315 804 10.1126/science.1137016 17289988

[B4] Jeoh T Ishizawa CI Davis MF Himmel ME Adney WS Johnson DK Cellulase digestibility of pretreated biomass is limited by cellulose accessibility Biotechnol Bioeng 2007 98 112 122 10.1002/bit.21408 17335064

[B5] Johnson DK Elander RT Pretreatments for enhanced digestibility of feedstocks Biomass recalcitrance: Deconstructing the plant cell wall for bioenergy 2008 Oxford: Wiley-Blackwell 436 453

[B6] Grethlein HE The effect of pore size distribution on the rate of enzymatic hydrolysis of cellulosic substrates Nat Biotechnol 1985 3 155 160 10.1038/nbt0285-155

[B7] Schell DJ Farmer J Newman M McMILLAN JD Dilute-sulfuric acid pretreatment of corn stover in pilot-scale reactor Appl Biochem Biotechnol 2003 105 69 85 10.1385/ABAB:105:1-3:69 12721476

[B8] Lloyd TA Wyman CE Combined sugar yields for dilute sulfuric acid pretreatment of corn stover followed by enzymatic hydrolysis of the remaining solids Bioresour Technol 2005 96 1967 1977 10.1016/j.biortech.2005.01.011 16112484

[B9] Eggeman T Elander RT Process and economic analysis of pretreatment technologies Bioresour Technol 2005 96 2019 2025 10.1016/j.biortech.2005.01.017 16112490

[B10] Aden A Ruth M Ibsen K Jechura J Neeves K Sheehan J Wallace B Montague L Slayton A Lignocellulosic Biomass to Ethanol Process Design and Economics Utilizing Co-Current Dilute Acid Prehydrolysis and Enzymatic Hydrolysis for Corn Stover NREL/TP-510-32438 NREL report 2002 Denver 1 154

[B11] Wikberg H Liisa Maunu S Characterisation of thermally modified hard-and softwoods by 13C CPMAS NMR Carbohydr Polym 2004 58 461 466 10.1016/j.carbpol.2004.08.008

[B12] Liitiä T Maunu S Hortling B Solid-state NMR studies of residual lignin and its association with carbohydrates J Pulp Pap Sci 2000 26 323 330

[B13] Robert D Lin S Dence C Carbon-13 Nuclear Magnetic Resonance Spectrometry Methods in Lignin Chemistry 1992 Berlin: Springer-Verlag 250 273

[B14] Kim DW Kim TS Jeong YK Lee JK Adsorption kinetics and behaviors of cellulase components on microcrystalline cellulose J Ferment Bioeng 1992 73 461 466 10.1016/0922-338X(92)90138-K

[B15] Mandels M Kostick J Parizek R The use of adsorbed cellulase in the continuous conversion of cellulose to glucose J Polym Sci C 1971 36 445 459

[B16] Mooney CA Mansfield SD Beatson RP Saddler JN The effect of fiber characteristics on hydrolysis and cellulase accessibility to softwood substrates Enzym Microb Technol 1999 25 644 650 10.1016/S0141-0229(99)00098-8

[B17] Zhang YHP Lynd LR Toward an aggregated understanding of enzymatic hydrolysis of cellulose: noncomplexed cellulase systems Biotechnol Bioeng 2004 88 797 824 10.1002/bit.20282 15538721

[B18] Puri VP Effect of crystallinity and degree of polymerization of cellulose on enzymatic saccharification Biotechnol Bioeng 1984 26 1219 1222 10.1002/bit.260261010 18551639

[B19] Bouchard J Leger S Chornet R Quantification of residual polymeric families present in thermo-mechanical and chemically pretreated lignocellulosics via thermal analysis Biomass 1986 9 161 171 10.1016/0144-4565(86)90086-7

[B20] Marx-Figini M The acid-catalyzed degradation of cellulose linters in distinct ranges of degree of polymerization J Appl Polym Sci 1987 33 2097 2105 10.1002/app.1987.070330621

[B21] Yachi T Hayashi J Takai M Shimizu Y Supermolecular structure of cellulose: stepwise decrease in LODP and particle size of cellulose hydrolyzed after chemical treatment. [Leveling-off degree of polymerization] J Appl Polym Sci, Appl Polym Sym 1983 Hiratsuka, Japan: Tokai Univ 325 343

[B22] Hallac BB Ragauskas AJ Analyzing cellulose degree of polymerization and its relevancy to cellulosic ethanol Biofuels Bioprod Biorefin 2011 5 215 225 10.1002/bbb.269

[B23] Lambert AG Davies PB Neivandt DJ Implementing the theory of sum frequency generation vibrational spectroscopy: a tutorial review Appl Spectrosc Rev 2005 40 103 145 10.1081/ASR-200038326

[B24] Barnette AL Bradley LC Veres BD Schreiner EP Park YB Park J Park S Kim SH Selective detection of crystalline cellulose in plant cell walls with sum-frequency-generation (SFG) vibration spectroscopy Biomacromolecules 2011 12 2434 2439 10.1021/bm200518n 21615075

[B25] LaComb R Nadiarnykh O Townsend SS Campagnola PJ Phase matching considerations in second harmonic generation from tissues: effects on emission directionality, conversion efficiency and observed morphology Opt Commun 2008 281 1823 1832 10.1016/j.optcom.2007.10.040 19343083 PMC2390911

[B26] Chandra R Ewanick S Hsieh C Saddler JN The characterization of pretreated lignocellulosic substrates prior to enzymatic hydrolysis, part 1: a modified Simons’ staining technique Biotechnol Prog 2008 24 1178 1185 10.1002/btpr.33 19194930

[B27] Chandra RP Ewanick SM Chung PA Au-Yeung K Del Rio L Mabee W Saddler JN Comparison of methods to assess the enzyme accessibility and hydrolysis of pretreated lignocellulosic substrates Biotechnol Lett 2009 31 1217 1222 10.1007/s10529-009-9993-5 19357812

[B28] Beldman G Voragen A Rombouts F Searle-van Leeuwen M Pilnik W Adsorption and kinetic behavior of purified endoglucanases and exoglucanases from Trichoderma viride Biotechnol Bioeng 1987 30 251 257 10.1002/bit.260300215 18581306

[B29] Kyriacou A Neufeld C Ronald J Effect of physical parameters on the adsorption characteristics of fractionated trichoderma reesei cellulase components Enzym Microb Technol 1988 10 675 681 10.1016/0141-0229(88)90059-2

[B30] Esteghlalian AR Bilodeau M Mansfield SD Saddler JN Do enzymatic hydrolyzability and Simons’ stain reflect the changes in the accessibility of lignocellulosic substrates to cellulase enzymes? Biotechnol Prog 2001 17 1049 1054 10.1021/bp0101177 11735439

[B31] Yu X Minor JL Atalla RH Mechanism of action of Simons’ stain Tappi J 1995 78 175 175

[B32] Lee D Yu AHC Wong KKY Saddler JN Evaluation of the enzymatic susceptibility of cellulosic substrates using specific hydrolysis rates and enzyme adsorption Appl Biochem Biotechnol 1994 45–6 407 415

[B33] Valdeir A Jack S Access to cellulose limits the efficiency of enzymatic hydrolysis: the role of amorphogenesis Biotechnol Biofuels 2010 3 4 14 10.1186/1754-6834-3-4 20178562 PMC2844368

[B34] Laureano-Perez L Teymouri F Alizadeh H Dale BE Understanding factors that limit enzymatic hydrolysis of biomass Appl Biochem Biotechnol 2005 121 1081 1099 15930583 10.1385/abab:124:1-3:1081

[B35] Chandra R Bura R Mabee W Berlin A Pan X Saddler J Substrate pretreatment: the key to effective enzymatic hydrolysis of lignocellulosics? Biofuels 2007 108 67 93 10.1007/10_2007_064 17530205

[B36] Nguyen QA Tucker MP Keller FA Beaty DA Connors KM Eddy F Dilute acid hydrolysis of softwoods Appl Biochem Biotechnol 1999 77 133 142 10.1385/ABAB:77:1-3:133

[B37] Tucker MP Kim KH Newman MM Nguyen QA Effects of temperature and moisture on dilute-acid steam explosion pretreatment of corn stover and cellulase enzyme digestibility Appl Biochem Biotechnol 2003 105–108 165 177 10.1385/abab:105:1-3:165 12721483

[B38] Sluiter A Hames B Ruiz R Scarlata C Sluiter J Templeton D Crocker D Determination of structural carbohydrates and lignin in biomass 2008 Denver: NREL Laboratory Analytical Procedure (LAP) 1 18

[B39] Evans R Wearne RH Wallis AFA Molecular weight distribution of cellulose as its tricarbanilate by high performance size exclusion chromatography J Appl Polym Sci 1989 37 3291 3303 10.1002/app.1989.070371202

[B40] Mormann W Michel U Improved synthesis of cellulose carbamates without by-products Carbohydr Polym 2002 50 201 208 10.1016/S0144-8617(02)00016-4

[B41] Park S Baker JO Himmel ME Parilla PA Johnson DK Research cellulose crystallinity index: measurement techniques and their impact on interpreting cellulase performance Biotechnol Biofuels 2010 3 1 10 10.1186/1754-6834-3-1 20497524 PMC2890632

